# Context-Guided SAR Ship Detection with Prototype-Based Model Pretraining and Check–Balance-Based Decision Fusion

**DOI:** 10.3390/s25164938

**Published:** 2025-08-10

**Authors:** Haowen Zhou, Zhe Geng, Minjie Sun, Linyi Wu, He Yan

**Affiliations:** College of Electronics and Information Engineering, Nanjing University of Aeronautics and Astronautics, Nanjing 210016, China; zhouhaowen2002@outlook.com (H.Z.); 18551013132@163.com (M.S.); wly5010106@nuaa.edu.cn (L.W.); yanhe@nuaa.edu.cn (H.Y.)

**Keywords:** synthetic aperture radar (SAR), ship detection, automatic object detection, deep learning, remote sensing

## Abstract

To address the challenging problem of multi-scale inshore–offshore ship detection in synthetic aperture radar (SAR) remote sensing images, we propose a novel deep learning-based automatic ship detection method within the framework of compositional learning. The proposed method is supported by three pillars: context-guided region proposal, prototype-based model-pretraining, and multi-model ensemble learning. To reduce the false alarms induced by the discrete ground clutters, the prior knowledge of the harbour’s layout is exploited to generate land masks for terrain delimitation. To prepare the model for the diverse ship targets of different sizes and orientations it might encounter in the test environment, a novel cross-dataset model pretraining strategy is devised, where the SAR images of several key ship target prototypes from the auxiliary dataset are used to support class-incremental learning. To combine the advantages of diverse model architectures, an adaptive decision-level fusion framework is proposed, which consists of three components: a dynamic confidence threshold assignment strategy based on the sizes of targets, a weighted fusion mechanism based on president-senate check–balance, and Soft-NMS-based Dense Group Target Bounding Box Fusion (Soft-NMS-DGT-BBF). The performance enhancement brought by contextual knowledge-aided terrain delimitation, cross-dataset prototype-based model pretraining and check–balance-based adaptive decision-level fusion are validated with a series of ingeniously devised experiments based on the FAIR-CSAR-Ship dataset.

## 1. Introduction

Synthetic aperture radar (SAR) is an advanced microwave remote sensing technology that actively emits electromagnetic pulses and processes backscattered signals to generate high-resolution imagery. Unlike optical cameras, SAR operates independently of sunlight and weather conditions, enabling all-day, all-weather Earth observation [1,2]. With the continuous development of Sentinel-1, Gaofen-3, and other satellite-borne SAR systems, massive and multi-resolution SAR images can be acquired. The unique architecture of SAR imaging comprises three critical stages, including data acquisition, signal processing and image formation. Firstly, the satellite or aircraft transmits microwave pulses toward Earth’s surface; then, the radar receiver records backscattered echoes from ground targets. The received echo signal undergoes range–Doppler processing and azimuth compression to achieve metre-scale resolution. Finally, the processed signal is used to output an amplitude/phase image with pixel values characterizing the surface features, where the high-value areas are strong reflectors, such as ships, metal structures, etc., and the low-value areas are smooth surfaces, such as the calm sea surface. Driven by satellite constellations like Sentinel-1 (ESA) and Gaofen-3 (China), multi-resolution SAR data now supports maritime monitoring at unprecedented scales. SAR ship detection has become a key application which facilitates military reconnaissance, search-and-rescue operations, and marine traffic management.

With advances in computing power, big data, and algorithms, deep learning-based object detection has emerged as a pivotal computer vision technique [3]. This progress extends to remote sensing image analysis, where context-driven detection in complex environments attracts growing research [4]. However, research on context-driven SAR target detection is still rare due to the lack of high-quality annotated images.

The public release of annotated satellite-borne SAR images has accelerated deep learning applications in SAR ship detection. Given ships’ small size and sparse distribution in SAR imagery, conventional anchor-based detectors generate excessive low-quality candidate boxes. Consequently, anchor-free detectors, which bypass predefined anchor boxes to directly predict key target points, have gained prominence. Models like CornerNet [5], ExtremeNet [6], CenterNet [7], Objects as Points [8], FCOS [9], and FoveaBox [10] demonstrate superior efficiency and accuracy by eliminating redundant computations.

To address multi-scale detection challenges, it is a critical procedure to fuse low- and high-level features to expand the receptive field. Cui et al. [11] from the University of Electronic Science and Technology designed a dense attention pyramid network, linking convolutional attention modules across hierarchical feature maps to enhance positional and semantic feature extraction. Deng et al. [12] from the National University of Defense Technology introduced a multi-receptive-field extractor with ReLU and activation modules, generating candidate regions at intermediate layers for adaptive ship matching. Fu et al. [13] the Institute of Space and Astronautical Information proposed a feature balancing network combining anchor-free detection with attention-guided pyramid fusion, significantly improving small-ship recognition.

**Figure 1:**
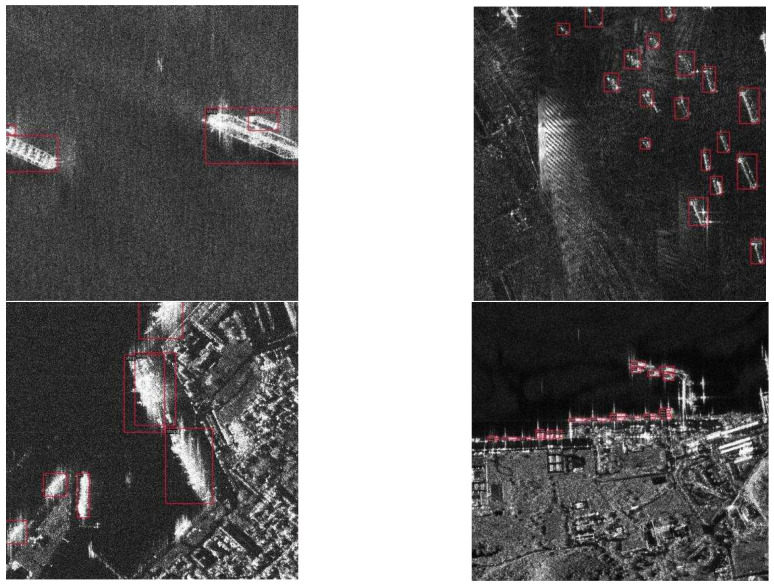
Comparing the scale variation in SAR images, where the red square marks the ship target.

**Figure 2:**
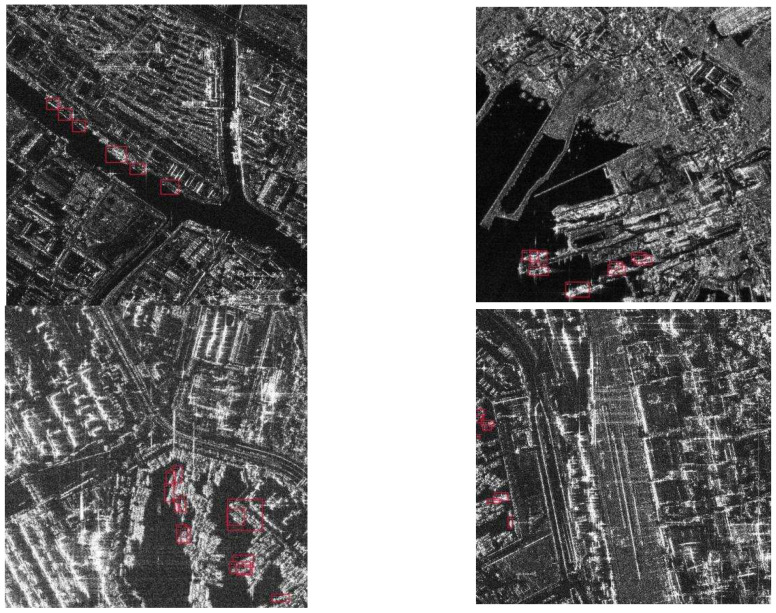
Complex interference in inshore scenarios, where the red square marks the ship target.

Despite the breakthrough of deep learning-based SAR ship detection methods in natural scenes, SAR ship detection still faces serious challenges:

1. Significant ship scale diversity, as shown in Figure 1. Ship targets in SAR images have huge size variations, ranging from small fishing boats, with dozens of pixels, to large freighters and oil tankers coexisting, featuring hundreds of pixels. This intra-class size variation stems from the diversity of imaging distance, ship tonnage, and orientation, making it difficult for a single model to accurately detect targets of different scales at the same time. This can very easily result in the omission of small targets or the inaccurate localization of large targets.
2. Complex interference in inshore scenarios, as shown in Figure 2. There are a large number of strong scattering man-made targets (e.g., cranes, oil storage tanks, breakwaters) with similar scattering characteristics to ships and land background clutter in inshore areas such as harbours, islands, etc., resulting in a surge of false alarm rate, especially in densely moored shoreline areas small ships are close to.

Aiming at the problems of ship size diversity and complex interference in inshore scenarios, this paper proposes a multi-model training strategy that integrates prior knowledge about harbour layout and ship prototype. The core idea of this strategy is to utilize the prior knowledge of harbour layout, such as the geospatial information of ports and berths, etc., so that the model can focus on the areas where ships could probably be present, thus suppressing the false alarm caused by the discrete feature clutter in the harbour area, and at the same time leverage the prior knowledge of the ship prototype provided by the auxiliary dataset for model pretraining, such as the geometrical shape feature of the typical ship categories (e.g., bulk carriers, container ships, oil tankers, etc.), to detect out-of-library ships whose features match those of the prototypes, but whose size is different.

The main contributions of this paper are as follows:

1. A training strategy for SAR ship detection with harbour layout and ship prototype prior knowledge is proposed. The harbour layout and ship prototype are taken as key prior knowledge and integrated into the deep neural network training process, which significantly improves the model’s ability of discrimination when dealing with ship size diversity and inshore complex background.
2. An adaptive decision-level fusion strategy is proposed based on dynamic confidence threshold selection. A novel weighted fusion mechanism mimicking the “president–senate” check–balance (PSCB) principle is proposed, where a dominant leading model (the president) and the committee of expert models (the senate) are designated based on their performance in model pretraining. The experimental results show that by employing the proposed strategy, the mAP compared to the dominant model can be improved by up to 3.8% when the ratio of training data is 50%.

The subsequent contents of this paper are organized as follows: Section 2 discusses the related research to this paper; Section 3 details the proposed multi-model training strategy that integrates harbour layout and ship prototype prior knowledge; Section 4 describes the datasets, evaluation metrics and experimental settings, and conducts an exhaustive analysis of comparative experimental results; and Section 5 concludes the full paper and looks forward to the direction of future research.

## 2. Related Work

Relevant prior work includes SAR ship detection based on FCOS, SAR ship detection based on ATSS, TOOD, PP-YOLOE and PP-YOLOE+.

### 2.1. SAR Ship Detection Based on FCOS

Currently, most mainstream target detection models are anchor-based, facing problems such as difficult dealing with targets with large-scale differences, especially small targets, imbalanced positive and negative samples, large computational complexity, etc. Z. Tian et al. from the University of Adelaide proposed a Fully Convolutional One-Stage Object Detector (FCOS) [9], adopting anchor-free, multilevel prediction with FPN, a centre-ness branching strategy used to get rid of the complex computation and hyperparametric tuning caused by the anchor-based algorithm, and achieved better performance than the anchor-based detection networks such as RetinaNet, YOLOm and SSD. Since then, FCOS has been widely used in the field of SAR ship detection.

Z. Sun et al. from National University of Defense Technology proposed a CP-FCOS ship detection model [14] to optimize the position regression branch features in the FCOS network by using a category-position (CP) module, which has a better detection performance compared to the Faster-RCNN, RetinaNet, and FCOS. X. Zhao et al. from University of Chinese Academy of Sciences proposed an R-FCOS [15] by introducing ship aspect angle and rotate regression branch to regress-oriented bounding boxes (OBBs) for arbitrary-oriented ship detection, and by proposing the use of ratio-ness branch to alleviate the effects of feature misalignment problem, achieving mAP@0.5 90.65% in the SSDD+ dataset. L. Zhou et al. from University of Electronic Science and Technology proposed a LASDNet [16], which achieved mAP@0.5 86.02% with a computational complexity of 1.01 GFLOPs. S. Yang et al. from China University of Petroleum proposed an Improved-FCOS [17] to extract effective features and collect global context through a multilevel feature attention mechanism. They proposed a feature refinement and reuse module with two stages to refine small-ship features, designed a head improvement module to optimize the methods for classification and localization of ship targets, and a modified varifocal loss to better train the classification branch, which was more accurate and robust on the SAR-Ship-Dataset, HRSID, and LS-SSDD-v1.0 dataset. D. Zhang et al. from the Army Engineering University proposed an end-to-end anchor-free rotate detector OFCOS [18] by proposing a feature-enhanced feature pyramid network (FE-FPN) to enhance the significance of object features, and by constructing a regression branch with orientation characterization capability to better describe the orientation of objects and a centre-to-corner bounding box prediction strategy to improve the accuracy of object localization. The model is validated on the publicly available dataset HRSC2016 and the self-constructed dataset RS-Ship and achieves 13.01% and 9.84% improvement compared to FCOS. The above study shows that the FCOS model performs well in the field of SAR ship detection and can be used as a basic model for related research.

### 2.2. SAR Ship Detection Based on ATSS

S. Zhang et al. from the Chinese Academy of Sciences analyzed the difference between anchor-free detector and anchor-based detector in positive and negative sample selection and proposed an adaptive training sample selection (ATSS) algorithm [19]. ATSS automatically calculates the positive and negative samples based on the target features, which reduces the hyperparameters required for sample definition. Thys far, there are few studies on SAR ship target detection based on ATSS. Q. Zhang et al. from Xi’an University of Electronic Science and Technology proposed a dual-scale feature-fusion segmentation network, called DRSNet [20], which uses a Gaussian-distribution-based ATSS (Ga-ATSS) to produce positive samples according to the shape information of ships for the training of the bounding-box detection module (DetHead). This algorithm achieves AP 67.5% and 68.1% on the SSDD dataset and the Instance-RSDD dataset, respectively, and the recall rises by 1.3%.

### 2.3. TOOD, PP-YOLOE and PP-YOLOE+

There is a task conflict problem between classification and localization in target detection. To further improve the detection accuracy and overcome the conflict between classification and localization, task-aligned one-stage object detection (TOOD) [21] is proposed, which provides a better balance between learning task interactivity and task-specific functionality through task-aligned head (T-Head) and learns alignment more flexibly through task-aligned predictor (T-aligned predictor). It also proposes task alignment learning (TAL), which, during training, through a designed sample assignment scheme and task-aligned loss, explicitly finds the best anchor points that are closer to the two tasks to achieve the alignment of target classification and localization.

PP-YOLOE [22] improves the detection head based on TOOD and proposes efficient task-aligned head (ET-head) to improve both speed and accuracy. PP-YOLOE adopts ESE instead of layer attention in TOOD, which simplifies the alignment process of classification branch. PP-YOLOE chooses varifocal loss (VFL) and distribution focal loss (DFL) for the learning of classification and localization, respectively. Unlike Quality Focal Loss (QFL), VFL uses a target score to measure the weight loss of positive samples, an approach that allows the model to focus more on high-quality samples than those of low quality during training. Similarly, both QFL and VFL use the IoU-aware Classification Score (IACS) as the target for prediction, which allows for the efficient learning of the joint representation of classification scores and localization quality estimates, resulting in a high degree of consistency between training and prediction.

PP-YOLOE+ [23] makes improvements in three aspects based on PP-YOLOE: the pretraining strategy, model architecture optimization, and vertical scene adaptation. PP-YOLOE+ introduces the large-scale target detection dataset Obj365 for the first time to carry out pretraining, and adjusts the parameter initialization strategy to reduce the training cycle in downstream task fine-tuning, which significantly improves the model’s feature extraction capability and convergence speed. Meanwhile, PP-YOLOE+ improves the end-to-end reasoning speed by optimizing image scaling, normalization and other preprocessing, and by implementing TAL (Dynamic Label Assignment Algorithm) through lightweighting. In addition, PP-YOLOE+ proposes rotating box detection PP-YOLOE-R [23] and small-target detection PP-YOLOE-SOD [24] for specific scenarios to realize the model architecture extension.

## 3. Methods

**Figure 3:**
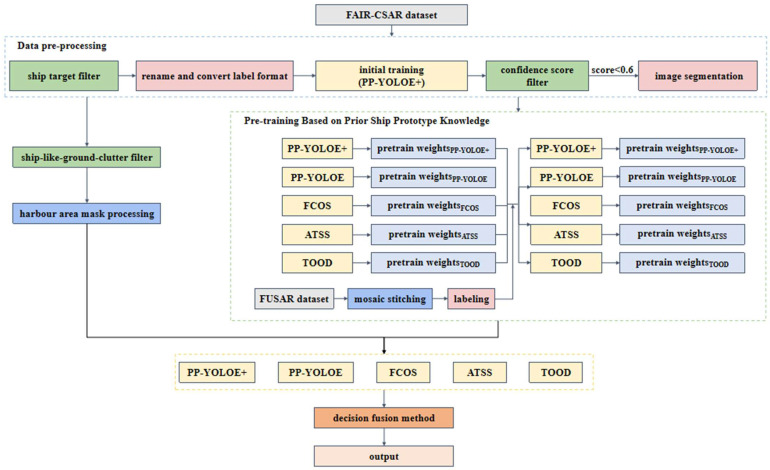
Structure of proposed model.

In this paper, we propose a multi-model training strategy that integrates harbour layout and ship prototype prior knowledge. It contains three main components, and its specific structure is shown in Figure 3. The first part is pretraining based on prior ship prototype knowledge, which is one of the key innovations of this paper. By utilizing four types of ships in the FUSAR dataset to pretrain the neural network to correctly recognize the morphology of ships, the small-target detection accuracy in complex maritime environments can be significantly improved, the specific details of which are shown in Section 3.2. The second part is harbour area mask processing, which is detailed in Section 3.3. By utilizing the prior knowledge of the harbour layout to generate a land mask for terrain delimitation, the neural network is prevented from misclassifying discrete ground clutter as ship targets, thus achieving a reduction in the false alarm rate, which is a major challenge in existing maritime surveillance systems. The third part is Adaptive Multi-model Decision-Level Fusion Processing, detailed in Section 3.4, where the results generated by multiple expert models are fused via dynamic confidence threshold selection, and a Soft-NMS-based Dense Group Target Bounding Box Fusion (Soft-NMS-DGT-BBF) algorithm is proposed to address the problem of overlapping target bounding boxes. Compared with the traditional static fusion strategy, the proposed strategy dynamically weights the detection results of different models according to the real-time target size and retains all high-quality detection boxes through a novel weighted fusion mechanism based on the president–senate check–balance, thus further improving the detection accuracy.

### 3.1. Pre-Processing of the FAIR-CSAR Dataset

**Figure 4:**
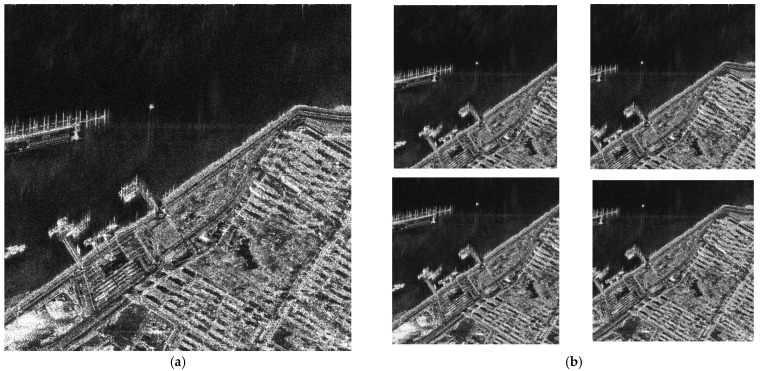
Image segmentation instances. (**a**) Original image; (**b**) example image slices generated from original image.

The FAIR-CSAR-V1.0 dataset is a fine-grained target dataset of SAR imagery made public by Y. Wu of the Chinese Academy of Sciences in 2025 [25]. The FAIR-CSAR dataset is constructed based on 175 views of the complete Gaofen-3 Class 1 single-view complex-value imagery products, covering airports, oil refineries, ports, and river channels in 32 regions around the world. The dataset covers 5 main categories and 22 subcategories, including 7 subcategories of ship targets, such as bulk carriers, warships, etc. Taking into consideration the superiority in image quality, annotation accuracy, and sample diversity of the FAIR-CSAR-V1.0 dataset over the other widely used datasets, in this paper, we carry out SAR ship target detection experiments based mainly on this dataset. Since the ship size information is not available, the images containing only ship targets are unanimously labelled as “ship”. Leveraging the PaddleDetection Toolbox, the PP-YOLOE+ model is utilized to conduct preliminary training and testing with the aim of screening out hard samples, i.e., images that are difficult to detect (confidence score less than 0.6). To reduce the negative impact of inshore clutter and improve the detection performance, the hard samples are magnified by a scaling factor and cropped into a couple of overlapping slices of size L × L with a predesignated step size approximately L/5 for further processing. An example of the image segmentation is shown in Figure 4.

### 3.2. Pretraining Based on Prior Ship Prototype Knowledge

**Figure 5:**
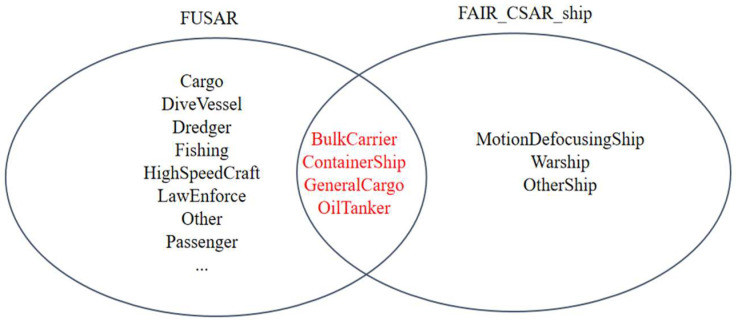
Comparison between ship types included in FUSAR dataset and those in FAIR-CSAR-Ship dataset, and the red text indicates the types of ships included in both datasets.

The FUSAR dataset is a high-resolution SAR-AIS-matched ship detection and recognition dataset made public by Hou et al. of Fudan University in 2020 [26], which contains a total of 126 GF-3 scenarios, covering a variety of scenarios such as the sea, land, coast, rivers, islands, etc. The FUSAR dataset contains more than 5000 slices of ships with AIS information, as well as strong scatterers, bridges, coastal land, islands, sea and land clutter samples. The ship types are diverse, with 15 major categories and 98 subcategories of ships, almost covering all types of ship targets, as shown in Figure 5. In order to improve the recognition ability of neural network on SAR ship targets in FAIR-CSAR-Ship dataset, and to improve the detection accuracy in different scenarios, this paper utilizes four types of ships in FUSAR dataset, namely, bulk carriers, container ships, general cargo ships, and oil tankers, to conduct pretraining on the four types of models with the prior knowledge of the SAR ship prototype, so as to train the ability of neural network to correctly recognize the morphology of different types of ships, and to further improve the accuracy when locating ship targets in complex scenes.

**Figure 6:**
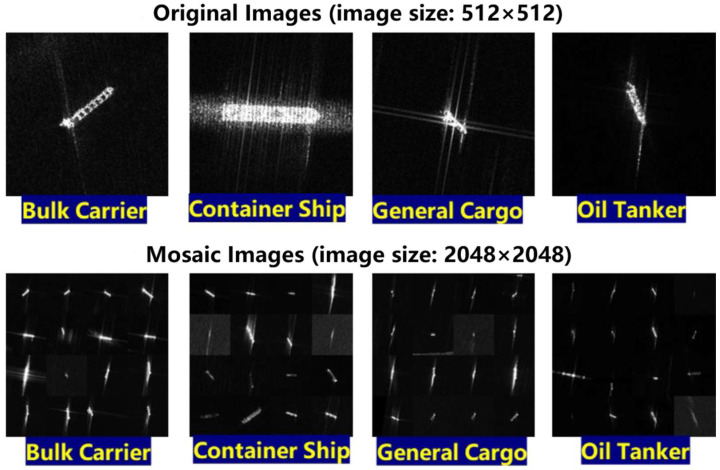
Mosaic images obtained by stitching SAR image chips corresponding to four types of ships into FUSAR dataset in groups of 16 (4 × 4).

To account for all the possible morphologies of the ships in the scenes of the FAIR-CSAR-Ship dataset, we searched diligently for SAR image chips containing bulk carriers, container ships, general cargo ships, and oil tankers with different sizes, orientations, and resolutions in the FUSAR dataset. Some representative mosaic images, obtained by stitching the SAR image chips corresponding to four types of ships in the FUSAR dataset in groups of 16 (4 × 4), are illustrated in Figure 6. Then, labelling is used to label ship targets in mosaic images; the targets in the synthetic mosaic images are annotated in the format of COCO; and the target name is unified as “ship” and then used to support the pretraining of diverse expert models for ship detection.

### 3.3. Harbour Area Mask Processing

**Figure 7:**
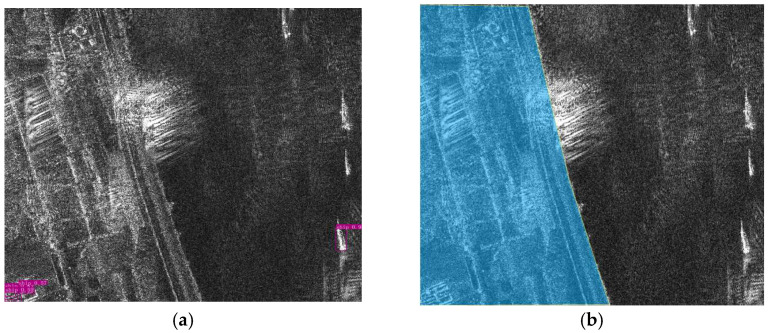
An example of harbour area mask processing under the assumption that the probability that a ship is present in any region on the water surface follows uniformly distribution. (**a**) Original image. The ground clutter at the lower left corner is misclassified as a ship target during the initial training. (**b**) Masked image. The false alarms are eliminated by employing the terrain delimitation mask, where the blue area refers to the terrain delimitation mask.

SAR image inshore ship detection is susceptible to the influence of discrete clutter in the harbour area, resulting in a large number of false alarms. In order to solve this problem, this paper adopts the method of harbour area mask processing. The specific steps are as follows: (1) screen out the hard samples featuring high false alarm rates in the initial training stage; (2) generate a mask for terrain delimitation based on the prior knowledge of the harbour layout, so that the models selectively focus on specific regions where ships are most likely to be present while the negative impact of inshore clutters is minimized. Depending on the type of harbour being considered (harbour of refuge, navy base, commercial harbour, and fishing harbour), the layout of the harbour usually varies greatly. To simplify the problem, it is assumed in this work that the probability that a ship is present in any region on the water surface follows uniformly distribution, and this simplification applies only to land masking and does not model actual ship distributions. An example of harbour area mask processing is shown in Figure 7. Note that for specific harbour layouts, the prior knowledge regarding the berth, quay, etc., could be incorporated into the mask in the form of probability density function.

### 3.4. Adaptive Decision-Level Fusion Based on Dynamic Confidence Threshold Selection

**Figure 8:**
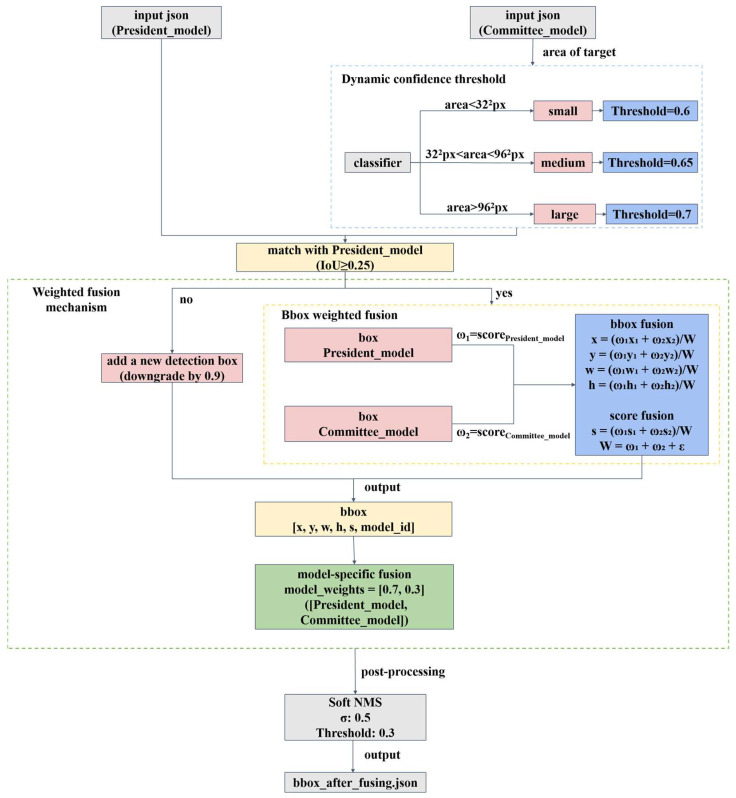
Structure of dynamic confidence threshold decision-level weighted fusion method.

Considering that each model has its unique inherent bias that brings favourable advantages over certain types of image samples and disastrous mistakes against other types of data, multi-model ensemble learning is always the best strategy. Therefore, this paper proposes an adaptive decision-level fusion framework, which consists of three components: dynamic confidence threshold assignment strategy based on the sizes of targets, weighted fusion mechanism based on president–senate check–balance, and Soft-NMS-based Dense Group Target Bounding Box Fusion (Soft-NMS-DGT-BBF). The specific framework is shown in Figure 8.

#### 3.4.1. Dynamic Confidence Threshold Assignment Strategy Based on the Sizes of Targets

Due to the diversity of ship target sizes in SAR images, small fishing boats with only a few dozen pixels and large cargo ships with hundreds of pixels may appear in the same image, and the detection of small ships is susceptible to the interference of high-intensity marine clutter, which makes it more difficult to detect them. In contrast, large ships are significant targets and less difficult to detect. Therefore, if the traditional static fusion strategy that relies on the screening of the detection boxes by confidence scores only is adopted, there is a great possibility that small ships will be missed. To account for the impact of target size on confidence score, dynamic thresholds are assigned according to the sizes of targets (small, medium, large), and the high-confidence target predictions made by different models are sifted out for decision-level fusion with the dominant model. Aiming at retaining small targets, expanding the candidate pool, and improving the recall rate in general, the confidence score thresholds for the three types of targets are set as 0.6, 0.65, and 0.7, respectively, based on the results of an extensive number of numerical simulations. If the confidence score of a detection box is lower than the threshold, its weight is set as 0, thus avoiding low-quality detections. Compared with the static weighting strategy that normalizes the mAP or accuracy of a single model on the test dataset to obtain fixed weights, the dynamic weighting strategy can adjust the weights according to the real-time output, which is more adaptable to complex scenarios.

#### 3.4.2. Weighted Fusion Mechanism Based on President–Senate Check–Balance

To combine the distinctive advantages of diverse model architectures, this paper adopts a novel weighted fusion mechanism mimicking the “president–senate” check–balance (PSCB) principle within the general framework of multi-model ensemble learning. The proposed fusion mechanism is a three-stage process: in Stage 1, a dominant leading model (the president) and the committee of expert models (the senate) are designated based on their performance in model pretraining; in Stage 2, if the prediction made by the leading model (like “nominations” made by the president) is approved by the committee, then the two prediction results will be fused according to weighting factors proportional to the confidence scores. However, if the committee propose other candidate prediction results that have not been nominated by the president model, these candidates will be added to the pool for further consideration but with a confidence score downgraded by 0.9, thus retaining the candidates favoured by the committee but disliked by the president. In Stage 3, the predictions made by the leading model and the committee are fused with weighting factors of 0.7 and 0.3, respectively. Compared with the traditional voting method fusion strategy, which treats each model equally under the false assumption that they have similar performance, the proposed method leverages the power of the committee to capture the targets missed by the president model and to support the high-confidence predictions made by the president model.

#### 3.4.3. Soft-NMS-Based Dense Group Target Bounding Box Fusion

**Figure 9:**
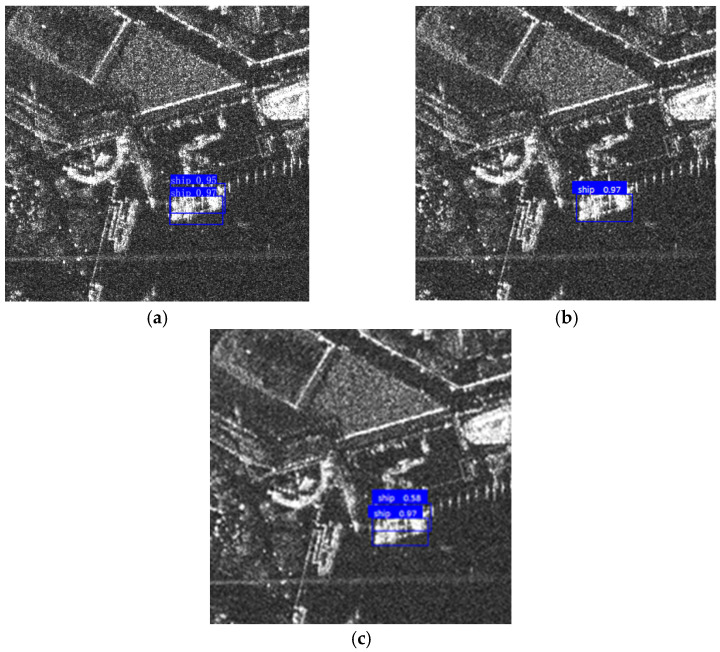
Illustration of advantage of proposed Soft-NMS-DGT-BBF algorithm over classic NMS method. (**a**) Original detection result, overlap ratio of detection boxes of two ships is 0.5. (**b**) Classic NMS processing results. Only the ship target with higher confidence is retained. (**c**) Soft-NMS-DGT-BBF processing results. Both ship targets are retained.

In the field of target detection, Non-Maximum Suppression (NMS) is commonly used to select the most promising prediction box among multiple candidate target boxes [27]. The NMS algorithm is able to filter and sort the target bounding boxes according to the confidence score and the overlap ratio, thus securing the best target bounding boxes based on the high-to-low confidence ranking. During the selection process, if the overlap ratio of two target bounding boxes is greater than a certain threshold (usually 0.5), the one with higher confidence is retained while the other is discarded, thus ensuring high-quality detection results. Although the NMS exhibits satisfactory performance for sparsely distributed objects with clear boundaries, using the NMS algorithm directly to the ship detection task illustrated in Figure 9b may lead to misdetections since the ships are densely distributed, partially occluded, and annotated with overlapping bounding boxes. Therefore, we propose a Soft-NMS-based Dense Group Target Bounding Box Fusion (Soft-NMS-DGT-BBF) algorithm specifically for the detection problem involving dense group targets belong to the same category based on the degree of overlap and the confidence score. The essence of the Soft-NMS-DGT-BBF algorithm is as follows: if there are two targets of the same category overlapping with each other, the detection box with the highest score is retained, while the scores of the other overlapping boxes are weighted by a Gaussian attenuation factor according to the formula in (1) as

(1) $$s_{i}=s_{i}e^{-\frac{IoU{\left(M,b_{i}\right)}^{2}}{\sigma }},\forall b_{i}\notin D$$

where M is the bounding box with the highest score, $b_{i}$ is the bounding box with score slightly below the highest score, D is the final set of test results, $s_{i}$ is the score to be updated in each iteration, and *σ* is a hyperparameter be set based on the density map of the images of interest [28]. It can be seen that the score attenuation is proportional to the *IoU* between *b_i_* and *M*, where *IoU* is defined as

(2) $$IoU=\frac{area(B_{p}\cap B_{gt})}{area(B_{p}\cup B_{gt})}$$

where $B_{p}$ and $B_{gt}$ represent the prediction bounding box and the ground truth bounding box, respectively. For the dataset considered in this paper, the overlap threshold is set to 0.3 empirically, while the Gaussian attenuation factor σ is set as 0.5. As shown in Figure 9c, the Soft-NMS-DGT-BBF algorithm correctly retains both ship targets in the original image, while the classic NMS processing method mistakenly kept one of the ships and neglected the other.

## 4. Experiment

Based on the performance evaluation results for dozens of representative model architectures in model pretraining, we consider 3 scenarios: (1) PP-YOLOE+ as the dominant leading model (the president), with FCOS, ATSS, and TOOD as the committee of expert models (the senate); (2) PP-YOLOE as the leading model, with FCOS and TOOD as the committee; (3) TOOD as the leading model, with FCOS andATSS as the committee. Section 4.1 compares the main stream SAR ship detection datasets that have been widely used in the field of deep learning-based SAR ship detection, where we explain why the FAIR-CSAR-V1.0 dataset has been chosen exclusively as the major dataset for the experiments in this section over the other widely used datasets (see Section 3.1 for details regarding image pre-processing). Section 4.2 introduces the performance evaluation metrics considered in this paper. Section 4.3 describes in detail the experimental platform and the hyperparameter settings of different models during the experiments. Section 4.4 quantitatively analyzes the performance enhancement brought by the delimitation masks generated based on the prior knowledge regarding the harbour, the pretraining procedure based on the FUSAR dataset, and the adaptive decision-level fusion framework.

### 4.1. Dataset

Until now, more than 10 public SAR ship datasets have been used for deep learning-based SAR ship detection and recognition research. Several early datasets, such as SSDD/SSDD+ [29], SRSDD-SAR [30], FUSAR-Ship, etc., are small datasets and a lack of diversity in image resolution and bands, which are prone to overfitting problems during deep learning training. Although many models exhibited excellent performance on the small datasets mentioned above, their detection performances decrease sharply when faced with out-of-library ship targets. In 2024, Nankai University in China constructed several small SAR ship datasets, including AIR-SAR-Ship [31], HRSID [32], SSDD, etc., and standardized them to form the first COCO-level large-scale multi-class SAR target detection dataset SARDet-100K, providing a new benchmark for researchers in the field of deep learning-based SAR ship detection [33]. The FAIR-CSAR dataset released by Chinese Academy of Sciences in 2025 [25] is the largest SAR target detection dataset (see Section 3.2) with the most detailed annotations, which include information regarding both the sensor (platform velocity, operating frequency, look direction, nominal resolution, etc.) and the object (category, subcategory, altitude/incidence angle, and key-points).

The widely used public datasets for SAR ship detection are summarized in Table 1, which covers four aspects: resolution (the highest resolution of the dataset); size (sample size in the dataset); images (the number of images); instances (the number of ships or instances). FAIR-CSAR dataset is selected as the benchmark dataset for this paper because of its superior performance in all aspects compared with other datasets.

### 4.2. Evaluations Metrics

The class-wise mean average precision (mAP) is used to quantify the detection performance of the proposed target detection framework. The performance tradeoff between the precision, which is defined as the ratio of true positives in all detections, and the recall rate, which is defined as the ratio of true positives in all the ground truths, is described by the precision–recall (P-R) curve. Assuming that the expression of the P-R curve is p(r), AP and mAP are obtained by Equation (3) and Equation (4), respectively, as

(3) AP=∫p(r)dr

(4) $$mAP=\frac{1}{N}\sum_{i=1}^{N}AP_{i}$$

where N is the number of classes. Despite of our willingness to thoroughly investigate the ship classification problem, we do not think it is appropriate to proceed without information on the size, orientation, and velocity of each ship object in the FAIR-CSAR dataset. Meanwhile, the resolution of the SAR images in the FAIR-CSAR dataset (1 m) is not fine enough to support accurate ship size and orientation estimation. Therefore, only the detection accuracy against a single target (ship) is considered in this paper, and the values of *mAP* and *AP* are the same.

Although mAP@0.5 is adopted in many research papers in academia as the performance metric, mAP@0.25 is usually chosen in industry for practical SAR ship detection systems and commercial SAR data products since it is a more reasonable metric given the far-from-satisfactory quality of the existing satellite-borne SAR images. For example, the offshore and inshore ship detection performance metrics released by Capella space to appeal to the potential paying customers are summarized in Table 2. It could be seen that the AP@0.25 for inshore vessels is only 0.74, while the AP@0.25 for small offshore vessels with a length between 15 m and 45 m is only 0.7. Therefore, in this paper, we present detection performance for both AP@0.25 (practical standard adopted in the industry) and AP@0.5 (benchmark for theoretical studies in academia).

**Figure 10:**
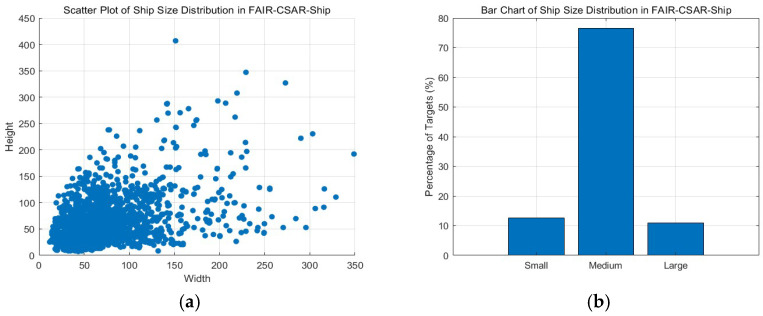
The ship target size distribution of FAIR-CSAR-ship measured by the width and height of the bounding box enclosing the morphological blob. (**a**) Scatter plot of ship size distribution in FAIR-CSAR-Ship, where the coordinates are in pixels; (**b**) bar chart of ship size distribution in FAIR-CSAR-Ship. The definitions for small, medium, and large objects are as follows: small objects have an area less than 1024 (32 px × 32 px), medium objects have an area between 1024 (32 px × 32 px) and 9216 (96 px × 96 px), and large objects have an area greater than 9216 (96 px × 96 px).

Figure 10 shows the distribution of sizes in the FAIR-CSAR-Ship dataset, where the size of a ship is measured by the target bounding box enclosing the morphological blob. It can be seen that this dataset is characterized by a variety of ship sizes, including small, medium, and large ship targets. Since statistics show that the number of medium-sized ship targets in this dataset is the largest, we set “area = medium” in the experimental process for an optimum performance.

### 4.3. Experimental Settings

The experiments are carried out on a computer with NVIDIA RTX A4000 GPU with CUDA 12.3. The PaddleDetection toolbox is used to carry out PP-YOLOE+-, PP-YOLOE- and TOOD-based experiments, while the MMDetection toolbox is used to carry out FCOS- and ATSS-based experiments. The specific hyperparameter settings, such as max-epochs, learning rate scheduler, etc., are summarized in Table 3. Standard Albumentations-library based image preprocessing and augmentation methods are also adopted, which include both pixel-level and spatial-level transforms.

### 4.4. Comparative Experiments

To begin, the dominant leading model (the president) and the committee of expert models (the senate) are designated based on their performance on the classical ship detection dataset HRSID. Table 4 summarizes the detection results of five detection models (PP-YOLOE+, PP-YOLOE, FCOS, ATSS, and TOOD) on the HRSID dataset. From the experimental results, it can be seen that all the selected models pass the basic AP threshold of 85% for SAR image detection. Since PP-YOLOE+ and PP-YOLOE exhibit better performance than the other models, they are designated as the president model and the vice president (VP) model, respectively.

To demonstrate the performance enhancement brought by the delimitation masks generated based on the prior knowledge regarding the harbour, the pretraining procedure based on the FUSAR dataset, as well as the adaptive decision-level fusion framework, experiments are carried out based on the pre-processed FAIR-CSAR-Ship dataset, the results for which are summarized below.

#### 4.4.1. Effectiveness of the Delimitation Masks

The experimental results are shown in Table 5, demonstrating the effectiveness of the delimitation masks based on the prior knowledge regarding the harbour. The enhanced PP-YOLOE+ achieves an AP@0.25 of up to 97.0% (with 70%/30% of the data for training/test), which is the highest accuracy among the selected models.

**Figure 11:**
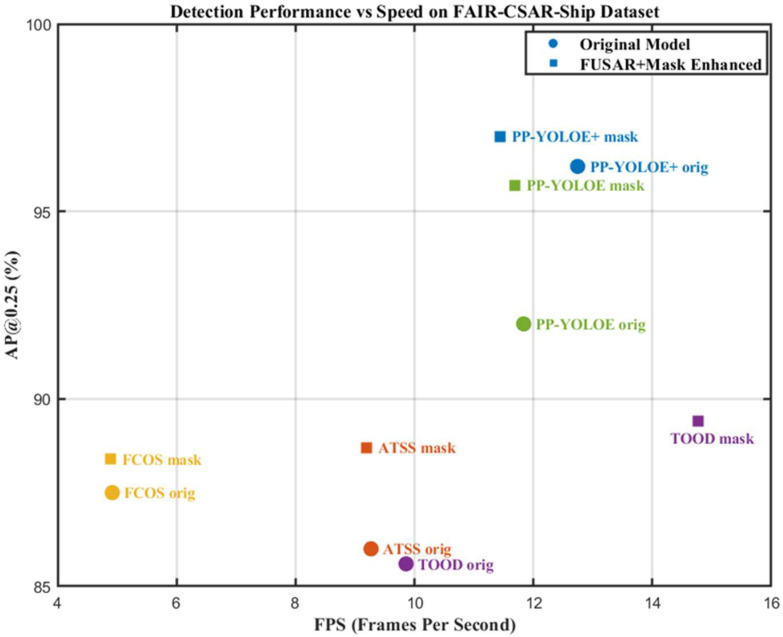
Performance versus speed on FAIR-CSAR-Ship dataset; 70%/30% of data for training/test.

Table 6 lists the comparison of FLOPs (Floating Point Operations) of the five models. It could be seen that when the image size is 1024 px × 1024 px, TOOD has the highest FLOPs of 0.403 T, while PP-YOLOE and PP-YOLOE+ have relatively small FLOPs of 0.125 T and 0.126 T, respectively. The FLOPs values indicates that all five models are light-weight models suitable for online training with out-of-distribution samples manually labelled by the operator in the deployment environment. Figure 11 shows the performance versus speed on the FAIR-CSAR-Ship dataset. It can be seen that although the enhanced TOOD has a significant advantage over the other four models in terms of reasoning speed, the AP@0.25 of it is not as good as that of PP-YOLOE and PP-YOLOE+, which is only 89.4%. When the two factors of reasoning speed and detection accuracy are taken into account, the performance of PP-YOLOE+ is significantly better than that of the other four models, being able to reach 11.4340 FPS and AP@0.25 97.0% after the enhancement processing.

#### 4.4.2. Effectiveness of the FUSAR-Based Pretraining

**Figure 12:**
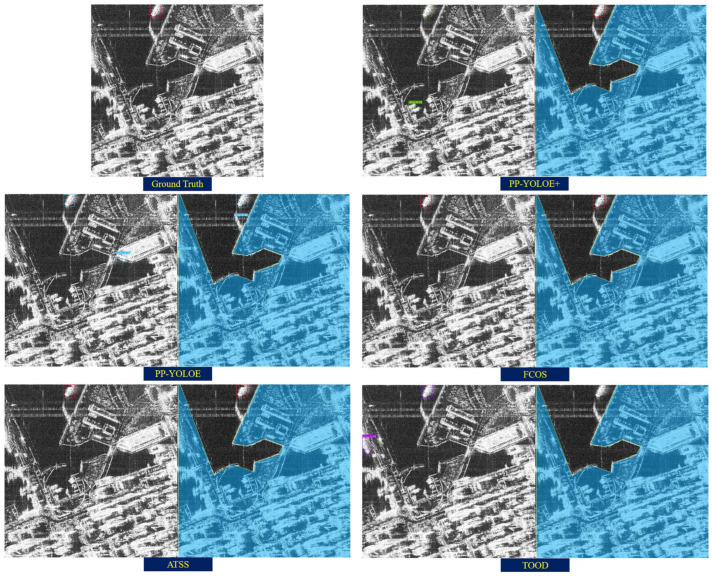
A typical scenario where simple terrain delimitation mask based on the uniform distribution assumption succeeded in false alarm elimination regarding inland discrete clutters, where the blue area refers to the terrain delimitation mask.

To evaluate the effectiveness of FUSAR-based Pretraining, two inshore scenes containing a large number of ground target-like clutter are chosen as the test samples. Figure 12 shows the detection results of the five models, respectively. For each pair of inference results, the left and the right figure are the detection results based on the original image and the masked image, respectively. It can be seen from Figure 12 that by applying a simple terrain delimitation mask based on the assumption that the probability that a ship is present in any region on the water surface follows a uniform distribution, the false alarms caused by inshore discrete ground clutters are reduced successfully.

**Figure 13:**
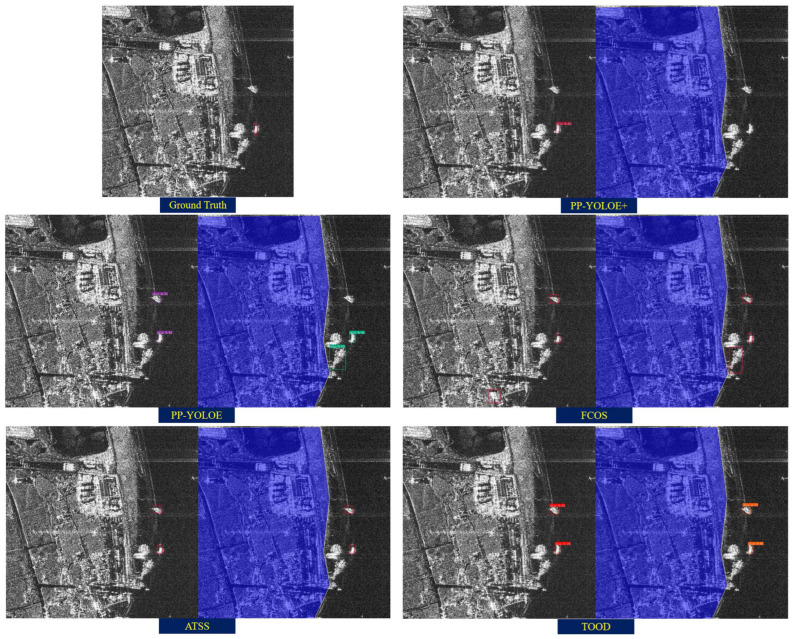
A typical scenario where simple terrain delimitation mask based on the uniform distribution assumption failed in isolating coastal ship-like clutters, where the blue area refers to the terrain delimitation mask.

However, to suppress ship-like clutters on water surface (which could be considered as a side-effect for FUSAR-based Pretraining), more sophisticated masks are required. For example, as shown in Figure 13, a near-coast ship-like clutter is mistaken as a ship target. To solve this problem, the best strategy is joint optical-SAR image analysis. Since SAR images usually come with the GPS information, the matching optical and multi-spectrum remote sensing data is actually free to pull from open sources like the Copernicus Data Space Ecosystem. Although the data update frequency could be in the range of days/weeks, fortunately the harbour of interest and the ship-like clutters around it are very unlikely to change overnight. By utilizing the stable context information contained in the optical image acquired in favourable-light conditions, SAR ship detection performance in poor light conditions would be further enhanced.

#### 4.4.3. Effectiveness of the Adaptive Decision-Level Fusion Framework

The effectiveness of the proposed adaptive decision-level fusion framework based on three pillars (i.e., the dynamic confidence threshold assignment strategy based on the sizes of targets, the weighted fusion mechanism based on president–senate check–balance, and the Soft-NMS-DGT-BBF post-processing method) is evaluated. Specifically, the great impact of model role assignment on the proposed adaptive decision-level fusion framework is evaluated with a series of comparative experiments, which shows that for a capable leader model, which resembles a wise president, decision fusion based on the suggestions made by the committee models only leads to slight performance improvement; but for a mediocre leader model, which resembles a mediocre VP stumbling into the presidential role due to luck, the contribution made by the committee is significant. The fused detection results are shown in Table 7. The results show that when PP-YOLOE+ acts as the president model, the contribution made by the committee in the decision fusion process is neglectable. However, when the less capable model PP-YOLOE is used as the dominant model, AP@0.25 and AP@0.5 are improved by up to 1.5% and 1.2% (with ratio of training data of 50%, MaxDets of 50), respectively; when TOOD is used as the dominant model, AP@0.25 and AP@0.5 are improved by up to 3.0% and 3.8% (with ratio of training data of 70%, MaxDets of 50), respectively.

The experimental results show that the proposed fusion strategy can effectively improve the detection accuracy, especially in the case that the president model is not well suited for the task at hand. Since a model is always selected into the office based on its performance in the training stage rather than in real-life deployment, which could be full of out-of-distribution samples, we prove that a committee made by expert models could effectively compensate for the uncertainties and improve the overall detection performance via check-and-balance systems.

**Figure 14:**
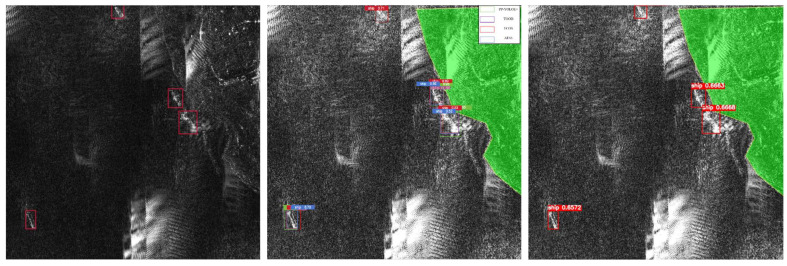

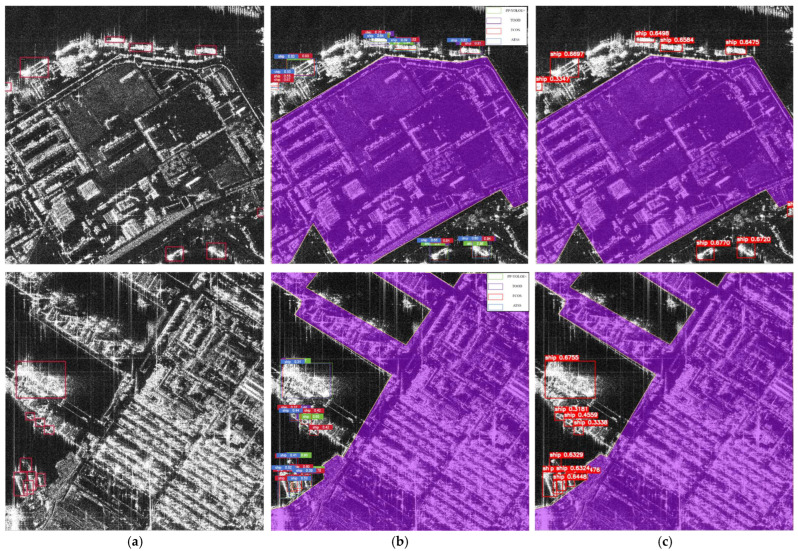
Results of fusion method of the same image when the president model is PP-YOLOE+ and the committee models are FCOS, ATSS and TOOD, with 70%/30% of the data for training/test, where the green and purple areas refer to the terrain delimitation mask. (**a**) Ground truth. (**b**) Predictions made by individual models. (**c**) Results generated by the proposed fusion strategy.

Figure 14 shows the fusion results when PP-YOLOE+ is the president model. It can be seen that the model PP-YOLOE+ with high accuracy can basically detect all ship targets; however, when the ship targets are incomplete (located at the edge of the image) or when the inshore ship targets are small in size and partially overlapped, the rest of the committee models are able to show some advantages. After the fusion is performed, the model is able to detect all ship targets with some improvement in the confidence score. The above results show that when less accurate models are chosen to fuse the president model, it can significantly compensate for the president model’s effectiveness in detecting specific targets.

## 5. Conclusions

To solve the problem in SAR ship detection caused by ship size diversity and the complex interference in inshore scenarios, this paper proposes a training strategy for SAR ship detection with harbour layout and ship prototype prior knowledge, and an adaptive decision-level fusion based on dynamic confidence threshold selection is utilized to fuse the detection results of several models to further improve the detection accuracy. The FUSAR dataset is selected to pretrain the neural network so that it has the ability to correctly recognize the ship prototype features. Then, the harbour area mask processing is conducted so that the model only focuses on specific regions where ships are most likely to be present, avoiding ground clutter from being mistaken as ship targets and achieving the purpose of reducing false alarms. In order to fully utilize the inherent characteristics of the different models, an adaptive decision-level fusion framework is proposed, which consists of three components: a dynamic confidence threshold assignment strategy based on the sizes of targets, weighted fusion mechanism based on president–senate check–balance, and Soft-NMS-based Dense Group Target Bounding Box Fusion (Soft-NMS-DGT-BBF). To verify the effectiveness of this strategy, five models are selected to train on the FAIR-CSAR-Ship dataset, i.e., PP-YOLOE+, PP-YOLOE, FCOS, ATSS, and TOOD. Experimental results show that by introducing the harbour layout and ship prototype prior knowledge can completely exclude ground false alarms; however, the performance is not satisfactory in dealing with some inshore ship-like clutter, which requires further optical-SAR image analysis. To validate the effectiveness of the adaptive decision-level fusion framework in improving detection accuracy, PP-YOLOE+, PP-YOLOE and TOOD are selected as the president models for decision-level fusion, respectively, and the results show that when the less capable model is used as the dominant model, a committee made up of expert models could effectively compensate for the uncertainties and improve the overall detection performance via check-and-balance measures.

Although the prior knowledge of the harbour can bring about significant improvements in the detection results, it is difficult for one research team at a university to cover more than a couple of harbours over the world, which limits the application of this method in open-world application to some extent. In this paper, due to the lack of knowledge about the specific distribution of the particular sea area or harbour, only the land area is considered for mask processing, without modelling the distribution of ships in the sea area; in addition, this paper adopts a manual annotation method rather than automatic annotation, which may have the risk of ambiguity of the sea–land boundary and the omission of the target due to the subjective error, and the scalability is poor. In the future, we will conduct in-depth research in the following aspects: adopting AI-assisted semi-automatic annotation as well as integrating open-source AIS geographic data for assisted inference to better utilize the scene prior knowledge to assist training.

## Figures and Tables

**Table 1:** Multi-dimensional comparisons of representative datasets for SAR ship detection.

Name	Source of SAR	Images	Instances	Resolution	Size
SSDD/SSDD+	RadarSat-2, TerraSAR, Sentinel-1	1160	2456	1 m–15 m	190–668
SAR-Ship-Dataset	Gaofen-3, Sentinel-1	43,918	59,535	3 m–25 m	256 × 256
HRSID	Sentinel-1	5604	16,951	0.5 m, 1 m, 3 m	800 × 800
SRSDD-SAR	Gaofen-3	666	2275	1 m	1024 × 1024
RSDD-SAR	Gaofen-3, TerraSAR	7000	10,263	2 m–20 m	512 × 512
FUSAR-Ship	Gaofen-3	126	6252	1.124 m × 1.728 m	512 × 512
SARDet-100K	Gaofen-3, Sentinel-1, TerraSAR, TanDEMX, HISEA-1, RadarSat-2	116,598	245,653	0.5 m–25 m	512 × 512
FAIR-CSAR	Gaofen-3	29,948	340,000	1 m, 5 m	1024 × 1024

**Table 2:** Performance metrics for SAR ship detection released on official website of Capella.

	Performance Metrics					AP Performance by Size Category				
	AP@0.25	F1@0.25	Precision	Recall	AP@0.25 by Image	XS <15 m	S 15–45 m	M 45–85 m	L 85–200 m	XL >200 m
Total	0.76 ± 0.02	0.74 ± 0.02	0.71	0.77	0.79 ± 0.02	0.69	0.76	0.89	0.91	0.91
Inshore	0.74 ± 0.02	0.73 ± 0.02	0.69	0.77	0.75 ± 0.02	0.69	0.76	0.89	0.90	0.89
Offshore	0.90 ± 0.01	0.86 ± 0.01	0.84	0.87	0.92 ± 0.01	0.26	0.70	0.95	0.95	0.95

**Table 3:** Experimental settings. “Initial LR” refers to initial learning rate. “Tr(IoU)” is IoU threshold. “DEF” means that default setting is used. “Norm.”, “RandRot”, and “Albmen.” represent normalization, random rotation, and Albumentations.

Model	Toolbox	Epochs	Initial LR	Scheuler	Tr (IoU)	Batch Size	Augmentation
PP-YOLOE+	Paddle Detection	80	1.25 × 10^−4^	DEF	0.7	8	Norm.
PP-YOLOE		240	1.25 × 10^−3^	DEF	0.7	8	Norm.
TOOD		100	1.25 × 10^−3^	DEF	0.6	4	Norm.
FCOS	MM Detection	100	5 × 10^−4^	Cosine AnnealingLR	0.5	2	Norm. + RandRot. + Albmen.
ATSS		180	1.5 × 10^−3^	Cosine AnnealingLR	0.6	2	Norm. + RandRot. + Albmen.

**Table 4:** The performance of the president model (PP-YOLOE+), the VP model (PP-YOLOE) and the committee models (FCOS, ATSS, TOOD) on the HRSID dataset (MaxDets = 100).

	PP-YOLOE+	PP-YOLOE	FCOS	ATSS	TOOD
AP@0.25 (%)	95.7	94.8	93.9	89.2	94.1
AP@0.5 (%)	94.3	92.6	90.4	85.6	91.9

**Table 5:** The results of different strategies on the FAIR-CSAR-Ship dataset.

Model	Evaluation Metrics	AP@0.25				AR@0.25–0.75			
	Ratio of Training Data	70%		50%		70%		50%	
	MaxDets	50	100	50	100	50	100	50	100
PP-YOLOE+	original	94.0	96.2	91.6	93.6	93.1	95.5	91.5	93.6
	FUSAR_pretrained	94.6 (+0.6)	96.7 (+0.5)	92.5 (+0.9)	93.9 (+0.3)	93.2 (+0.1)	95.4 (−0.1)	91.1 (−0.4)	93.5 (−0.1)
	FUSAR + mask	94.5 (+0.5)	97.0 (+0.8)	93.1 (+1.5)	94.0 (+0.4)	93.3 (+0.2)	96.0 (+0.5)	91.5	93.0 (−0.6)
PP-YOLOE	original	89.7	92.0	85.5	90.4	89.7	94.8	86.9	92.6
	FUSAR + mask	93.4 (+3.7)	95.7 (+3.7)	89.3 (+3.8)	93.3 (+2.9)	92.3 (+2.6)	95.5 (+0.7)	89.4 (+2.5)	93.9 (+1.3)
ATSS	original	83.6	86.0	77.7	80.6	84.1	88.2	78.2	82.0
	FUSAR + mask	86.4 (+2.8)	88.7 (+2.7)	81.1 (+3.4)	82.7 (+2.1)	85.8 (+1.7)	89.4 (+1.2)	81.4 (+3.2)	83.4 (+1.4)
FCOS	original	85.5	87.5	81.7	82.9	81.8	84.4	76.8	79.2
	FUSAR + mask	86.5 (+1.0)	88.4 (+0.9)	82.2 (+0.5)	84.3 (+1.4)	83.0 (+1.2)	84.9 (+0.5)	78.3 (+1.5)	80.0 (+0.8)
TOOD	original	83.6	85.6	79.3	81.9	79.2	83.0	75.8	80.0
	FUSAR + mask	87.7 (+4.1)	89.4 (+3.8)	85.7 (+6.4)	87.3 (+5.4)	82.8 (+3.6)	85.2 (+2.2)	81.3 (+5.5)	83.8 (+3.8)

**Table 6:** Comparison of flops for four models.

Model	FLOPs
PP-YOLOE+	0.126 T
PP-YOLOE	0.125 T
ATSS	0.179 T
FCOS	0.174 T
TOOD	0.403 T

**Table 7:** The results of decision fusion method on the FAIR-CSAR-Ship dataset.

President Model	Committee Model	MaxDets	Training Data Ratio	AP@0.25 (%)	AP@0.5 (%)
PP-YOLOE+	FCOS, ATSS, TOOD	100	70%	97.2 (+0.2)	95.0
			50%	94.1 (+0.1)	90.5 (−0.1)
		50	70%	94.6 (+0.1)	92.1 (−0.3)
			50%	932 (+0.1)	89.3 (−0.5)
PP-YOLOE	FCOS, TOOD	100	70%	96.2 (+0.5)	93.2 (+0.2)
			50%	94.2 (+0.9)	90.1 (+0.4)
		50	70%	94.0 (+0.6)	90.5 (+0.1)
			50%	90.8 (+1.5)	85.9 (+1.2)
TOOD	FCOS, ATSS	100	70%	90.4 (+1.0)	82.9 (+1.3)
			50%	88.0 (+0.7)	78.2 (+0.4)
		50	70%	90.7 (+3.0)	84.0 (+3.8)
			50%	88.2 (+2.5)	78.5 (+2.6)

## Data Availability

Some of the data are available upon request.
